# Koala ocular disease grades are defined by chlamydial load changes and increases in Th2 immune responses

**DOI:** 10.3389/fcimb.2024.1447119

**Published:** 2024-11-12

**Authors:** Samuel Phillips, Danielle Madden, Amber Gillett, Bonnie L. Quigley, Martina Jelocnik, Sankhya Bommana, Denis O’Meally, Peter Timms, Adam Polkinghorne

**Affiliations:** ^1^ Centre for Bioinnovation, University of the Sunshine Coast, Sippy Downs, QLD, Australia; ^2^ School of Science, Technology and Engineering, University of the Sunshine Coast, Sippy Downs, QLD, Australia; ^3^ Australia Zoo Wildlife Hospital, Beerwah, QLD, Australia

**Keywords:** *Chlamydia pecorum*, koala, ocular disease, disease progression, Th1/Th2 immune responses

## Abstract

**Introduction:**

This study employs bulk RNA sequencing, PCR, and ELISA assays to analyze the pathological factors affecting the outcomes of *C. pecorum* ocular infections in koalas. It investigates the immune responses and gene expression profiles associated with various stages of koala ocular chlamydiosis.

**Methods:**

A cohort of 114 koalas from Queensland, Australia were assessed, with 47% displaying clinical signs of ocular disease. Animals were classified into three cohorts: acute active disease (G1), chronic active disease (G2), and chronic inactive disease (G3), along with subclinical *Chlamydia pecorum* positive (H2) and healthy (H1) cohorts.

**Results:**

Analysis of clinical, microbiological, humoral immune and cellular immune biomarkers revealed varying chlamydial loads and anti-chlamydial IgG levels across disease grades, with a negative correlation observed between ocular chlamydial load and anti-chlamydial IgG. Koala ocular mucosa gene expression analysis from 27 koalas identified shared expression pathways across disease cohorts, with a significant upregulation of IFNγ expression and tryptophan metabolism in all disease stages.

**Discussion:**

These findings help elucidate immune response dynamics and molecular pathways underlying koala ocular chlamydiosis, providing insights crucial for disease management strategies.

## Introduction

The koala (*Phascolarctos cinereus*) is an arboreal, herbivorous marsupial and a globally recognized icon of Australian biodiversity. The term “koala” is believed to originate from a word in the Dharug language, spoken by the Indigenous peoples of the region between Parramatta and the Blue Mountains in New South Wales, meaning “no water.” In South East Queensland, koalas are referred to as “dumbirrbi” in the Jagera language, “marrambi” in the Yugarabul language, “borobi” in the Ugambeh language, and “dumbribbi” in the Turrbul language (Government, Q and S.a.I. Department of Enviroment, 2017). Unfortunately, koalas are in decline and were recently listed as an endangered species in New South Wales, Queensland, and the Australian Capital Territory (Quigley and Timms, 2020; Government, A and W.a.t.E. Department of Agriculture, 2022). Threats to the koala’s survival include urban expansion, habitat fragmentation, motor vehicle traumas, dog attacks and disease (Rhodes et al., 2011). Population modelling has suggested that control of disease is one of the most important strategies for returning peri-urban koala populations to stable levels (Rhodes et al., 2011).

The obligate intracellular bacteria *Chlamydia* is regarded as the most important pathogen contributing to the decline and long-term viability of koala populations (Quigley and Timms, 2020). Of the two species known to infect the koala, *Chlamydia pecorum* is the most pathogenic and prevalent (Nyari et al., 2017; Quigley and Timms, 2020), with records of prevalence as high as 80% in some populations (Quigley and Timms, 2020). Chronic *C. pecorum* infections in koalas can result in the development of cystitis, reproductive cyst development, and keratoconjunctivitis (Quigley and Timms, 2020).

Despite the devastating impact of this disease on koala populations, very little is known about the progression of ocular disease in koalas (Nyari et al., 2019; Quigley and Timms, 2021). Notably, observations seen in the koala closely match clinical observations of trachoma (Polkinghorne et al., 2013), also caused by infection with a related *Chlamydia* species (*C. trachomatis*) (Polkinghorne et al., 2013; Derrick et al., 2015). Trachoma research has shown that the disease begins with an acute symptomatic infection known as acute active conjunctivitis. Acute active conjunctivitis can clear naturally or progress further, developing into chronic active conjunctivitis. Chronic active conjunctivitis is marked by persistent conjunctival and eyelid inflammation and conjunctival hyperplasia, which is the biggest risk factor for scarring of the cornea resulting in blindness (Derrick et al., 2015). The microbiological and immunological mechanisms underlying disease progression remain an important knowledge gap in both humans and koalas (Mathew et al., 2013a; Wolle et al., 2021). Studies of trachoma indicate the involvement of multiple factors, including host genetics (Derrick et al., 2015), the innate and adaptive immune response in the conjunctiva (Burton et al., 2011) and epithelial cell responses effected by chlamydial infections (Rasmussen et al., 1997). Other contributing factors may include the composition of the ocular microbiome (Hu et al., 2010) and infection dynamics such as the number of repeat infections (Taylor et al., 2014) and the *Chlamydia* infectious load (Burton et al., 2003; Faal et al., 2006). Importantly, the most influential factors in worsening/progressing trichiasis is the frequency and duration of inflammation.

In koalas, research has suggested that a high *Chlamydia* load is associated with ocular disease progression (Nyari et al., 2017; Quigley and Timms, 2020). However, late stage chronic disease has been correlated with lower *Chlamydia* loads, indicating additional factors besides pathogen load are involved in pathogenesis (Nyari et al., 2017). In koala ocular disease, progression is determined by the number of repeat *Chlamydia* infections, koala retrovirus (KoRV) load and individual immune responses (Quigley et al., 2018a; Quigley and Timms, 2020).

Perhaps the most significant knowledge gap is our incomplete understanding of the koala immune response to *Chlamydia* infection. Preliminary studies of peripheral blood mononuclear cells have shown that interleukin-10 (IL10), interferon gamma (IFNγ), interleukin-17 (IL17) and tumor necrosis factor alpha (TNF-α) are significantly upregulated in the systemic immune response of diseased koalas (Mathew et al., 2013a; Mathew et al., 2013b; Mathew et al., 2014). To date, no study has compared the gene expression of healthy and diseased koalas at the conjunctiva.

Here, we use bulk RNA-seq, PCR and ELISA assays to characterize the pathological factors that influence the outcome of *C. pecorum* ocular infections in the koala. We show that higher chlamydial loads are associated with more severe ocular disease, particularly in the chronic active stage. IgG (*Chlamydia* EB specific) increases with disease severity, peaking in the chronic active stage (characterized by conjunctival hyperplasia and suppurative exudation), and decreases in the chronic inactive stage (little or no discharge and little evidence of active inflammation. Conjunctival hyperplasia and evidence of corneal scarring/opacity often present. We show that ocular mucosa IFNγ expression and tryptophan metabolism are significantly upregulated in all ocular disease stages compared to healthy controls, suggesting a consistent Th1-mediated immune response. These findings provide crucial insights into the dynamics of the immune response and molecular pathways underlying koala ocular chlamydiosis, paving the way for the development of appropriate intervention strategies (Khan et al., 2016; Phillips et al., 2019b).

## Methods

### Description of koalas and samples

**Figure 1:**
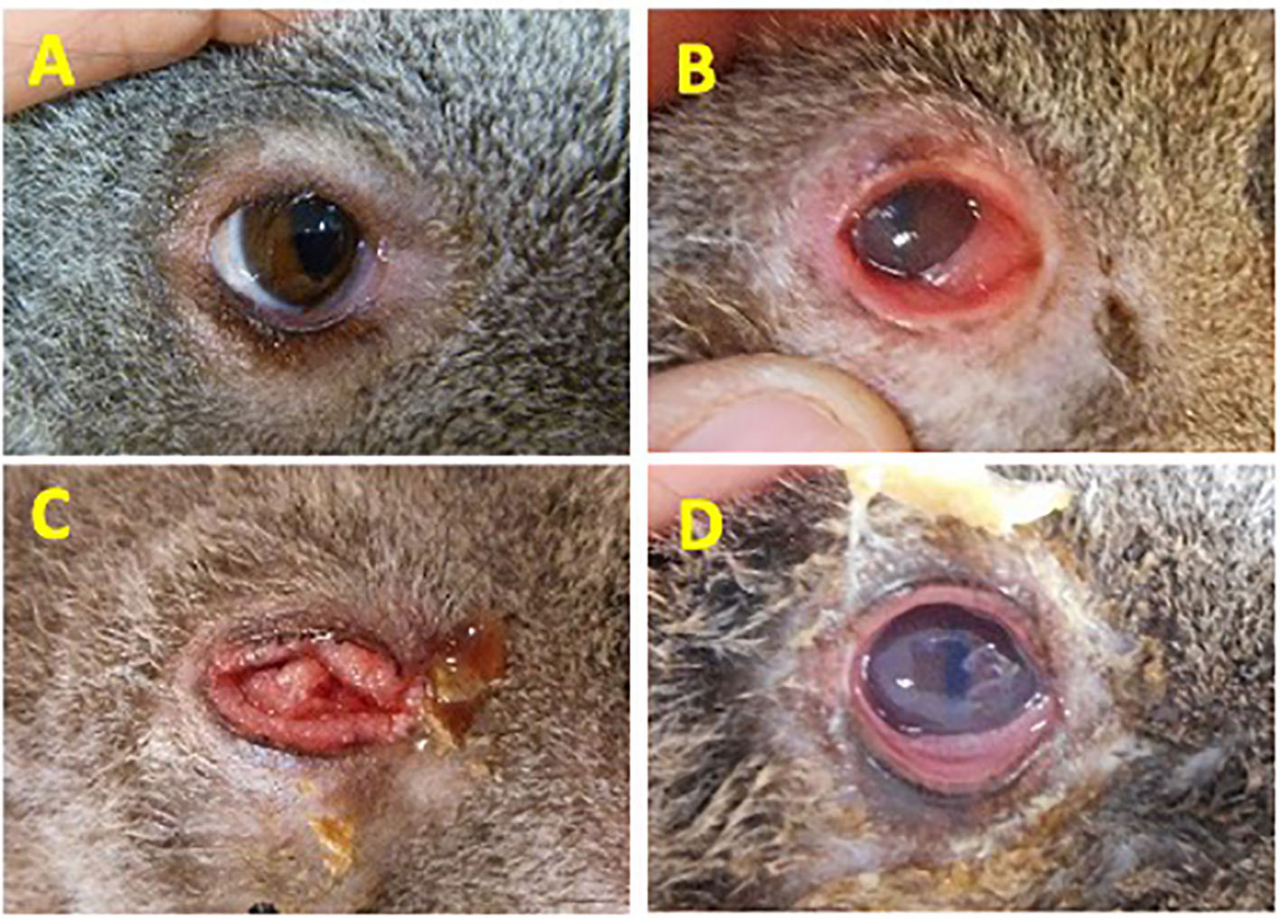
Disease grading criteria used to define the severity of ocular disease in koalas. **(A)** Healthy No clinical signs of disease, *C. pecorum* qPCR negative, cohort “Healthy (H1)” or *C. pecorum* qPCR positive, cohort “Healthy (H2)”; **(B)** Acute active disease, inflammation, and reddening (hyperaemia) of the conjunctiva. Absence of conjunctival hyperplasia though conjunctival oedema may be present, cohort “Grade 1 (G1)”; **(C)** Chronic, active disease, keratoconjunctivitis and inflammation, characterized by conjunctival hyperplasia, reddening (hyperaemia) and suppurative exudation. Corneal oedema may be present. Cohort “Grade 2 (G2)”; **(D)** Chronic inactive disease, little or no discharge and minimal evidence of inflammation. Conjunctival hyperplasia and/or corneal scarring/opacity often present. Cohort “Grade 3 (G3)”. Eye images shown are representative of animals in each cohort.

A total of 114 koalas presenting to Australia Zoo Wildlife Hospital (Beerwah, Queensland), Currumbin Wildlife Hospital (Currumbin, Queensland) and Moggill Koala rehabilitation Centre (Moggill, Queensland), each located in the southeastern state of Queensland, Australia were sampled and allocated into four disease cohorts based on the severity or absence of clinical signs of chlamydial disease (**Figure 1**). The assignment of animals in each cohort was made by a single experienced wildlife veterinarian from photographic evidence taken during a standardized veterinary assessment. Criteria for disease assessment were based on a previously described chlamydial ocular disease scoring system (Wan et al., 2011), with the criteria for Grade 2 (G2) and Grade 3 (G3) switched to improve the understanding of this scheme (allowing for disease progression to increase in disease grade 1–3). Ocular disease assessment was scored as follows: Healthy no clinical signs of disease, *C. pecorum* qPCR negative, cohort “Healthy (H1)” or *C. pecorum* qPCR positive, cohort “Healthy (H2)”. Acute active disease, inflammation, and reddening (hyperaemia) of the conjunctiva (absence of conjunctival hyperplasia though conjunctival oedema may be present), cohort “Grade 1 (G1)”. Chronic, active disease, keratoconjunctivitis and inflammation, characterized by conjunctival hyperplasia, reddening (hyperaemia) and suppurative exudation (corneal oedema may be present), cohort “Grade 2 (G2)”. Chronic inactive disease, little or no discharge and minimal evidence of inflammation. Conjunctival hyperplasia and/or corneal scarring/opacity often present, cohort “Grade 3 (G3)” (**Figure 1**).

For each animal, cotton-tipped swabs were used to sample the diseased conjunctiva (or left eye in a healthy animal) by ‘twirling’ the swab across the mucosal surface. To investigate possible concurrent chlamydial infections at other anatomical sites, urogenital swabs were collected from 96 koalas. All swabs were stored at 4°C prior to being resuspended in 500μl of PBS or RNAlater and stored at -80°C for later use (see **Supplementary Table S3** for sample and koala details).

Serum samples were also obtained from 65 koalas across the disease cohorts (40 diseased, 25 healthy). Blood samples were collected from the cephalic vein into EDTA collection tubes. Whole blood was then centrifuged, and separated plasma was transferred in fresh tubes and stored at -80°C use (see **Supplementary Table S3** for sample and koala details).

### Swab sample processing

DNA was extracted from ocular and urogenital swabs as previously described (Wan et al., 2011). Briefly, samples were vortexed vigorously for 5 minutes in PBS and centrifuged at 16,000g for 20 minutes. The pellet was resuspended in 50 µL of TE buffer with proteinase K and incubated at 56**°**C overnight, as previously described (Nyari et al., 2019). DNA was extracted using a QIAamp DNA kit (Qiagen, Germany) according to the manufacturer’s instructions and stored at -20**°**C for future use.

### *C*. *pecorum* detection

The presence of *C. pecorum* DNA was assessed and quantified utilizing a species-specific qPCR assay targeting a 209 bp fragment of the conserved hypothetical gene, as previously described (Jelocnik et al., 2017). Quantification was performed by comparison of the sample cycle threshold (Ct) to a standard curve produced using a serial dilution of known standards of DNA (see **Supplementary Table S3** for sample and koala details).

### Koala retrovirus subtype B detection

The presence of koala retrovirus (KoRV) RNA subtype B was assessed utilizing a subtype specific qPCR assay targeting a 304 bp fragment of the envelope gene, as previously described (Quigley et al., 2019).

### Anti-chlamydia IgG ELISA

*C*. *pecorum*-specific systemic IgG titres were determined from 65 koala plasma samples by ELISA assay targeting heat-inactivated, semi-purified *C. pecorum* koala strain MC/Marsbar elementary bodies, as previously described (Carey et al., 2010; Desclozeaux et al., 2017; Nyari et al., 2019) (see **Supplementary Table S3** for sample and koala details).

### RNA extraction and sequencing

For koala gene expression profiling, RNA extractions were performed on 27 ocular swabs. Extraction was performed as previously described (Mathew et al., 2013b) which included a vortex/centrifugation step to release koala cells and secretions from the mucosal swab into the RNALater suspension prior to kit extraction. RNA was then extracted with a RNeasy Mini Kit (Qiagen, Germany), according to manufacturer’s instructions, with the additional on-column DNase treatment. RNA-seq library construction and sequencing were performed by The Ramaciotti Centre (UNSW, Kensington, NSW) with TruSeq stranded mRNA chemistry on a NextSeq500 (Illumina, USA) (see **Supplementary Table S3** for sample and koala details).

### Transcriptome quality control and mapping to the koala genome

Sequence files for each sample were processed as previously described (Phillips et al., 2019a). Briefly, each sub-sequence file was concatenated into a single file and assessed for quality using the FastQC program (Adrews, 2010). Each sample was then mapped to the published koala annotated genome (GCF_002099425.1) using the sequence alignment program STAR (version 2.7.11b) with default settings. Read quantification was performed using HTseq (version 0.11.3) with the options -m intersection-non empty and -s reverse, utilising an adjusted GTF file from the published koala annotated genome (GCF-002099425.1) for annotation, and the subsequent count files combined in R (Team, R.C, 2018). Sequence reads have been deposited in the SRA database, BioProject identification number: PRJEB26467. This BioProject (PRJEB26467) is part of a larger study that includes 41 bio samples, however only 27 bio samples were utilised for this study, representing koala ocular transcriptomes.

### Statistical analysis

*C. pecorum* qPCR results and end point titre IgG ELISA results were compared utilising a Wilcoxon test with significant values denoted as follows; ns = p > 0.05, * = p <= 0.05, ** = p <= 0.01, *** = p <= 0.001, **** = p <= 0.0001. RNA sequencing read counts were analysed using a quasi-likelihood approach with a minimum read count of 10,000 across five samples. Differential gene expression (DGE) was compared between healthy koalas (10 koalas from groups H1 and H2) and each ocular disease grade (11 koalas from groups G1, G2 and G3). All statistical analyses were performed on the statistical platform R (version 4.1.2) utilising the packages EdgeR (3.36.0), statmod (1.4.37), ggplot2 (3.4.0), ggvenn (0.1.9) and tidyverse (1.3.2).

### Power analysis based on samples collected

We conducted power analyses using the ‘pwr’ package in R to assess the statistical power for each analysis based on the number of samples included. For the Wilcoxon test, with 114 samples, the analysis revealed that in a two-tailed test with a significance level of 0.05, the study would have a power of 0.96 for detecting a medium effect size. Additionally, for examining the correlation between chlamydial load and IgG levels, a power analysis with 65 samples showed that, in a two-tailed test with a significance level of 0.05, the power would be 0.99 for a large effect size and 0.69 for a medium effect size.

### Animal ethics

This study was considered and approved by the University of the Sunshine Coast (USC) Animal Ethics Committee (AN/S/15/36).

## Results

### Description of koala disease cohorts

A cohort of 114 koalas presenting to one of three wildlife hospitals in the southeastern state of Queensland, Australia were sampled for this study. At the time of admission, 54/114 koalas (47%) displayed clinical signs of ocular disease consistent with chlamydiosis. Following veterinary assessment, animals were assigned to the following cohorts: Grade 1 (G1) acute active ocular disease (17/114; 15%), Grade 2 (G2) chronic, active ocular disease (23/114; 20%) and Grade 3 (G3) chronic inactive ocular disease (14/114; 12%). The remaining 60/114 (53%) koalas displayed no clinical signs of disease (H). *C. pecorum* qPCR screening of the DNA extracted from conjunctival samples from the healthy ‘H’ koala cohort revealed that 33/60 (55%) koalas had detectable (2 - 275 gene copies/μL; IQR 7.7) *C. pecorum* DNA from their conjunctival swab. These animals were classified into a new cohort; subclinical *C. pecorum* positive (H2). The remaining 27 ocular *C. pecorum* negative animals with no signs of disease were assigned the cohort ‘H1’. KoRV subtype B positivity was also assessed from all 114 koalas from swab samples collected. Amplification of the *env* gene identified similar positivity of KoRV-B across all groups of koalas with 52%, 58%, 52%, 52%, and 64% of koalas infected in groups H1, H2, G1, G2 and G3, respectively.

To explore the connections between ocular infection and concurrent urogenital tract (UGT) infection, we evaluated UGT *C. pecorum* positivity in a subgroup of 96 koalas from cohorts H1 to G3 (18 swabs from group H1, 32 from H2, 14 from G1, 21 from G2, and 11 from G3) who underwent paired ocular and UGT swab collection. Of the 96 animals with paired ocular and UGT swabs, 59 (60.8%) had *C. pecorum* DNA detected at both sites, while 12 (12.3%) were qPCR negative at both sites. Seventeen animals (17.5%) were *C. pecorum* positive at the UGT site only, and eight (8.2%) at the ocular site only. When comparing the *C. pecorum* load at each, UGT swabs had a greater range and higher average *C. pecorum* load (+1.9 x 10^3^ copies/µL) than the ocular swabs (**Supplementary Table S1**).

### Ocular *C. pecorum* infectious load and anti-chlamydial IgG have a negative correlation

**Figure 2:**
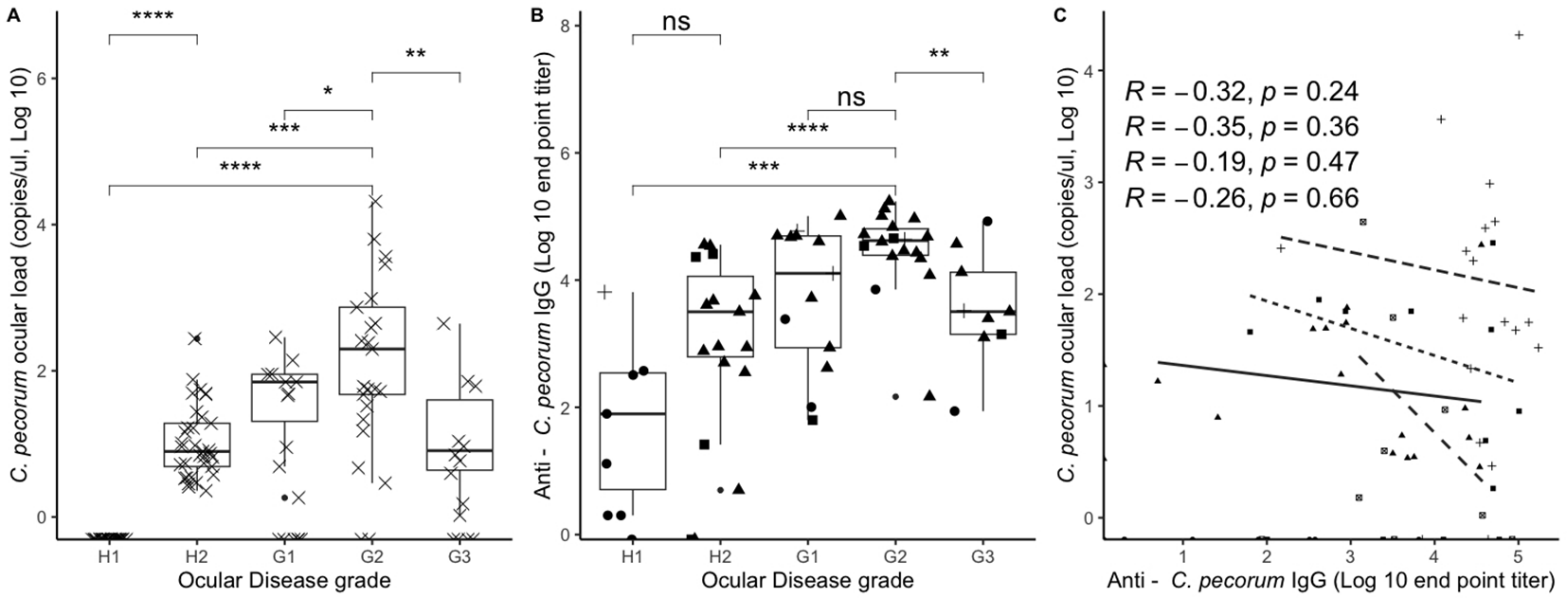
A boxplot and correlation analysis of chlamydial ocular load and anti *C. pecorum* IgG responses reveals significant differences between koalas in different ocular disease cohorts and a negative correlation between IgG and chlamydial load increases. **(A)** Chlamydial load by ocular disease grade; **(B)** anti *C. pecorum* IgG by ocular disease grade, shapes represent no infection as circle, ocular and UGT infection as triangle, ocular infection only as square, and UGT infection only as plus sign.; **(C)** Chlamydial load over anti *C. pecorum* IgG correlation, shapes and line type represent ocular disease grade H1 as circle (no line), H2 as triangle and solid line, G1 as square and small dash line, G2 as plus sign and large dash line and G3 as square with a cross and spaced out dashed line. Comparisons assessed by the nonparametric Wilcoxon test and spearman’s correlation test, with boxes corresponding to the first and third quartiles (the 25th and 75th percentiles) and whiskers representing the first and forth quartiles. Significant values are denoted as follows; ns = p > 0.05, * = p > 0.05, ** = p ≤ 0.01, *** = p ≤ 0.001, **** = p ≤ 0.0001. Figure was constructed using R studio.

*C. pecorum* screening revealed variation in the bacterial loads between the ocular disease cohorts. Of the *C. pecorum* positive koalas, H2 (23.9 ± 48.7 copies/µL) and G1 koalas (50.3 ± 74.7 copies/µL) had similar low *C. pecorum* loads. Animals in the G2 cohort were found to have the highest *C. pecorum* loads (1615.2 ± 4435.4 copies/µL) while the chlamydial loads in animals in the G3 cohort (43.8 ± 116.6 copies/µL) decreased to levels similar to that observed in H2 and G1 animals (**Supplementary Table S1**). *C. pecorum* loads in the G2 cohort were significantly different to *C. pecorum* loads in all other cohorts (H1, H2, G1 and G3) with p values of <0.000, <0.000, 0.014 and 0.003 respectively (**Figure 2A**).

The relationship observed between ocular infection load and disease grade was also observed in the host systemic IgG antibody titres from a subset of 65 koalas. Overall, systemic IgG levels in the disease cohorts showed a significant (p = 0.0001) increase in systemic IgG levels in koalas increasing from healthy (H1) to acute active disease (G2), then decreasing during chronic disease (**Figure 2B**). The presence of a concurrent urogenital infection did not change the level of systemic IgG antibody levels in any of the disease cohorts (H2, G1, G2 and G3) (**Figure 2B**), also a correlation analysis of cumulative chlamydial load (ocular and UGT) compared to systemic IgG antibody levels did not significantly alter the findings from ocular only (**Supplementary Figure S1**). Furthermore, there was a significant difference (p = 0.075) between active chronic (G2) and inactive chronic (G3) disease but not to active acute (G1) (p = 0.13). Although, there was a trend of less IgG in the active chronic (G1) diseased koalas.

Despite the healthy H1 cohort of koalas having no chlamydial infection (except one with a *C. pecorum* urogenital infection), all but one koala had anti-*Chlamydia* IgG antibodies detected, indicating that these koalas had previous infections or infections at anatomical sites not screened. The anti-chlamydial IgG response in the healthy *C. pecorum* negative (H1) cohort was lower but not significantly different from the response measured in the subclinical *C. pecorum* positive (H2) cohort (p=0.051) (**Figure 2B**). Surprisingly, there was a negative correlation observed between the ocular chlamydial load and anti-chlamydial IgG for all disease grades, indicating that as the koalas *Chlamydia* load decreases the anti-chlamydia IgG titres increase. However, none were significant and indicate that higher numbers of koalas are necessary to confirm these findings (**Figure 2C**).

### Assessment of overall genomic expression at the koala conjunctiva between healthy and diseased koalas

A genome-wide gene expression analysis was performed in koalas with acute or chronic, active, or inactive chlamydial ocular disease. This analysis included a total of 21 koalas, including six healthy *C. pecorum* negative (H1) koalas, four subclinical *C. pecorum* positive (H2) koalas, three acute active disease (G1) koalas, four chronic active disease (G2) koalas and four chronic inactive disease (G3) koalas.

Overall expression analysis from the five ocular disease cohorts revealed significant differences in global gene expression from koala conjunctival cells in each clinical disease grouping with a biological coefficient of variation of 0.583, considered acceptable given the outbred uncontrolled grouping of the koalas.

**Figure 3:**
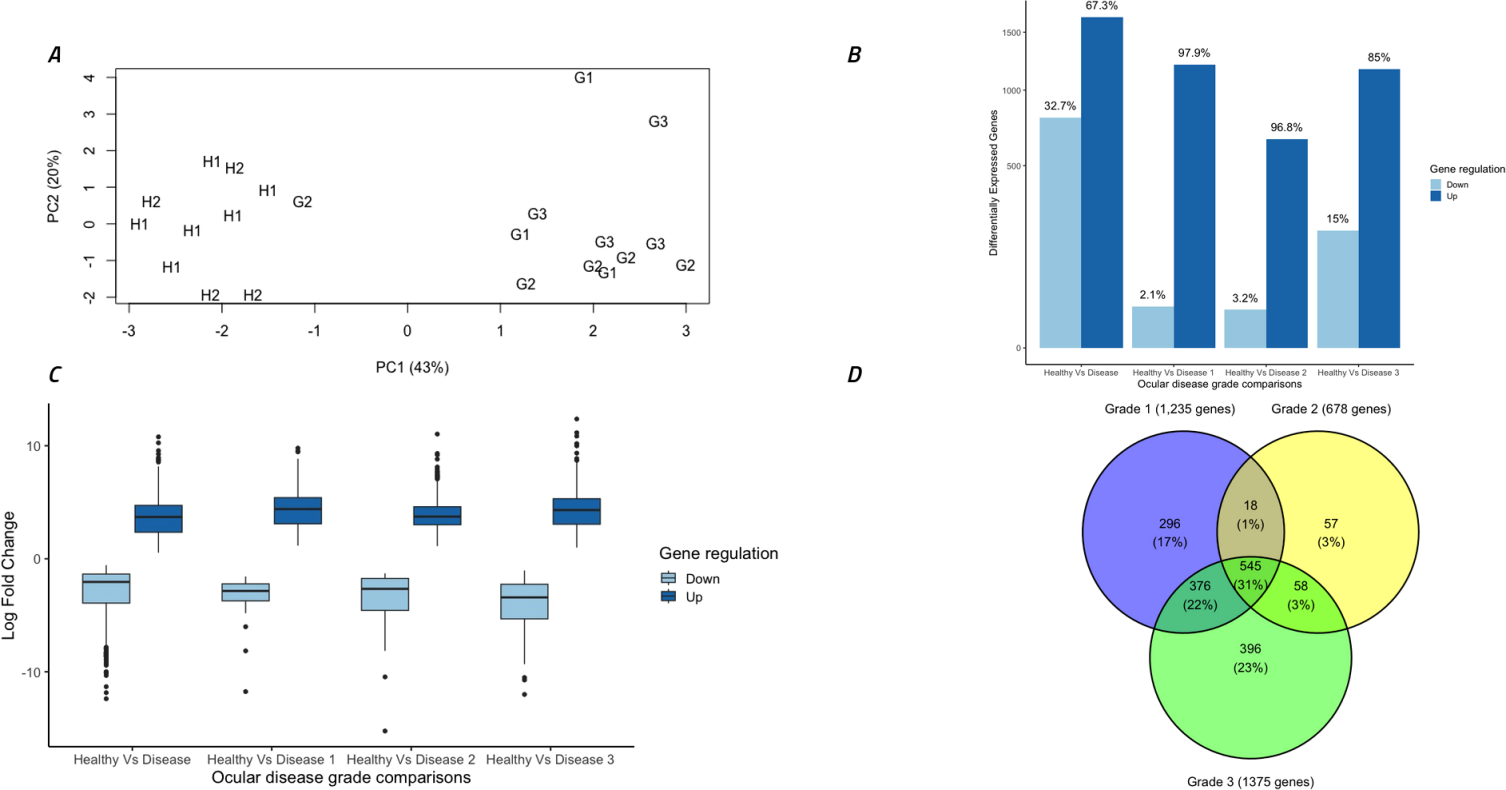
Overall analysis of gene expression data and differential gene expression comparisons between different disease grades. **(A)** Gene expression from 22 ocular samples by principal components analysis of all samples utilizing all normalized gene counts. Symbols indicate disease stage: H1 = healthy *C. pecorum* negative koalas, H2 = subclinical *C. pecorum* positive koalas, G1 = acute active disease koalas, G2 = chronic active disease koalas and G3 = chronic inactive disease koalas. **(B)** Total differentially expressed genes (DEG) (FDR <0.005) in each group separated into up and down regulated genes. **(C)** Comparison of the log fold changes in all DEGs, separated into up and down regulated genes. **(D)** Venn diagram indicating the number of DEGs between Healthy koalas (H1 and H2) and each ocular disease grade (G1, G2 and G3). Figure was constructed using R studio.

Principal components analysis of normalised gene expression counts determined that 43% of the variation between samples could be explained in the first component (PC1) and 20% could be explained in the second component (PC2) (**Figure 3A**). Strikingly, this separated all healthy koalas (H1 and H2) into one distinct cluster while 11 out of 12 of the diseased koalas (G1, G2 and G3) separated into another distinct cluster. A surprising result was that the single outlying G2 koala suffered from active bilateral conjunctivitis with severe ulceration of both eyes.

Differential gene expression analysis between ocular disease groups identified 2,446 (67% up-regulated) significantly (FDR <0.005) differentially expressed genes (DEGs) between healthy koalas (H1 and H2 combined, n=10) and diseased koalas (G1, G2 and G3 combined, n=12). There were no DEGs identified between cohorts H1 and H2, so these groups were combined for further analysis and termed “healthy koalas”. Comparison between healthy koalas and each disease cohort (G1, G2 and G3) separately identified 1,235 (98% up-regulated), 678 (97% up-regulated) and 1,375 (85% up-regulated) DEGs, respectively (**Figure 3B**). Down regulated genes accounted for 2%, 3% and 15% for each disease cohort respectively. Analysis of the extent of gene expression difference for each identified gene ranged from 1 to 12 log fold increase (average of 4 log fold increase) for up-regulated genes and a range of -1 to -15 log fold decrease (average of -3 log fold decrease) for down-regulated genes (**Figure 3C**).

Breaking down these overall cohort differences to identify overlaps in expression patterns within the disease states (G1, G2 and G3 separately compared to H1 and H2 combined), showed specific genes were significantly altered in expression within each stage of disease. Overall, 545 genes (31% of all genes) were significantly different between all stages of disease (G1+G2+G3) compared to healthy (H1+H2) (**Figure 3D**). Narrowing down the comparisons, disease cohorts G1 and G3 (which possessed lower *C. pecorum* loads and systemic IgG levels compared to disease cohort G2) shared 375 genes (22% of all genes) that were differentially expressed compared to healthy, but also contained 296 (17.0%) and 396 (23%) uniquely expressed genes, respectively (**Figure 3D**). Disease cohort G2 (which represented the highest *C. pecorum* loads and systemic IgG responses) was the least unique cohort, with only 57 (3%) DEGs specific to this disease grade (**Figure 3D**).

### Transcriptomic similarities between disease ocular cohorts

**Figure 4:**
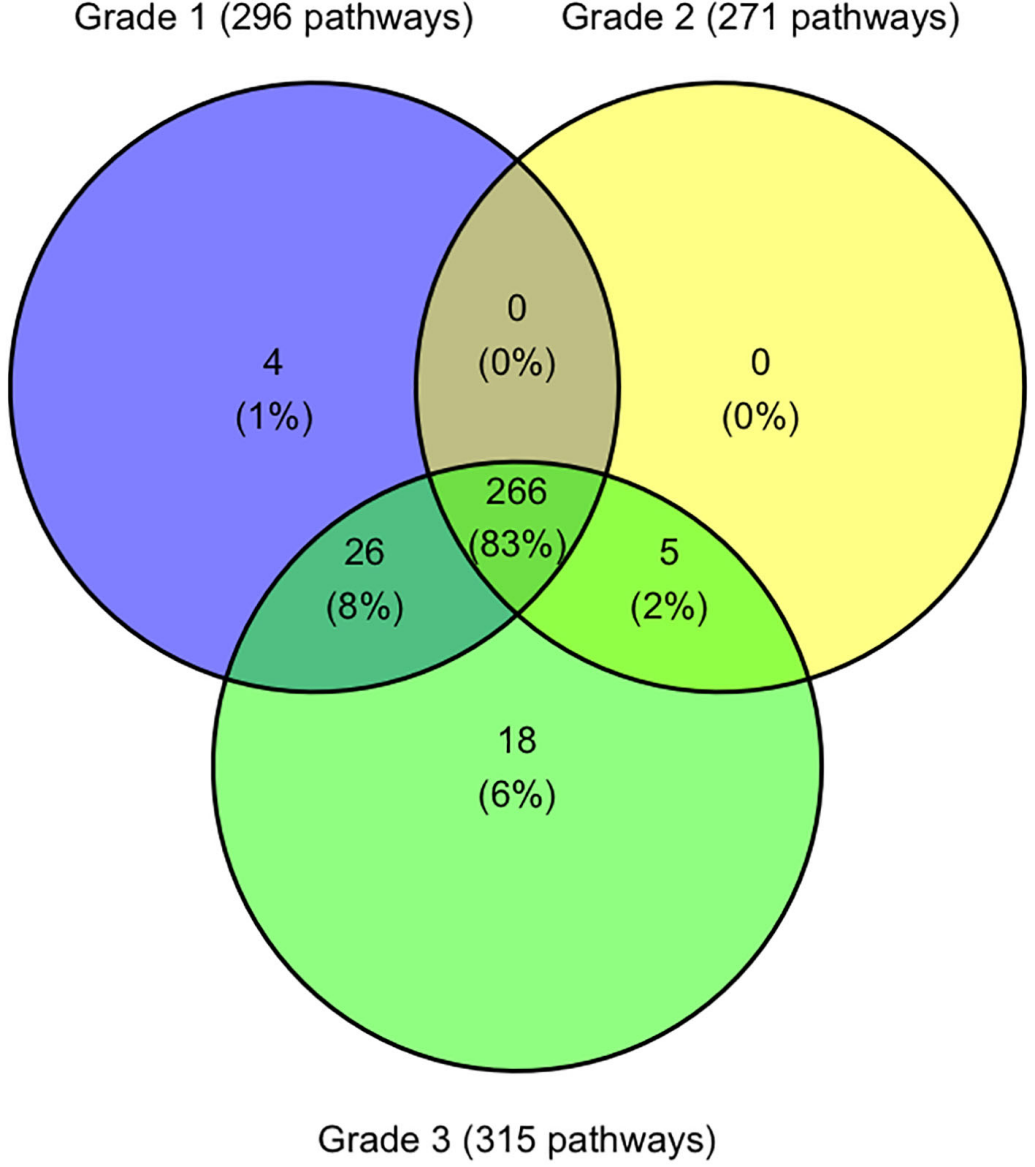
Venn diagram indicating the number of KEGG pathways shared and unique between koala ocular disease grades. Figure was constructed using R studio.

Analysis of all differentially expressed genes (DEGs) identified 1,747 unique genes with significantly altered expression between healthy koalas and one or more disease states, with 545 altered genes being shared across all three disease cohorts (G1, G2 and G3). Of these, 535 genes were identifiable with Entrez gene identifiers utilising the DAVID Bioinformatics database (10 genes not recognised) (Adrews, 2010; Sherman et al., 2022) and matched to 319 koala specific KEGG pathways (**Supplementary Table S2**). When assessed across the three disease cohorts, 83.4% (266/391) of pathways are shared across all disease grades (**Figure 4**). Five pathways were shared across disease states G2 and G3 only, including biosynthesis and metabolism pathways and the Oxidative phosphorylation pathway.

There were 26 pathways shared between disease states G1 and G3 which included p53 signalling pathways, RIG-I-like receptor signalling pathways and 13 different metabolism and synthesis pathways (**Supplementary Table S2**).

Four pathways were only identified in disease state G1 with the pathway “SNARE interactions in vesicular transport” being the only pathway involving multiple genes (syntaxin-11 and syntaxin-3) (**Supplementary Table S2**).

Disease state G3 had 18 unique pathways identified (**Figure 4**), with two pathways involving four genes, metabolism of xenobiotics by cytochrome P450 and fatty acid elongation (**Supplementary Table S2**). Other pathways with less involved genes include five different metabolism pathways, two degradation pathways and pathways involved in transportation and synthesis (**Supplementary Table S2**).

### Interferon gamma expression and tryptophan metabolism are upregulated in all ocular disease stages

**Figure 5:**
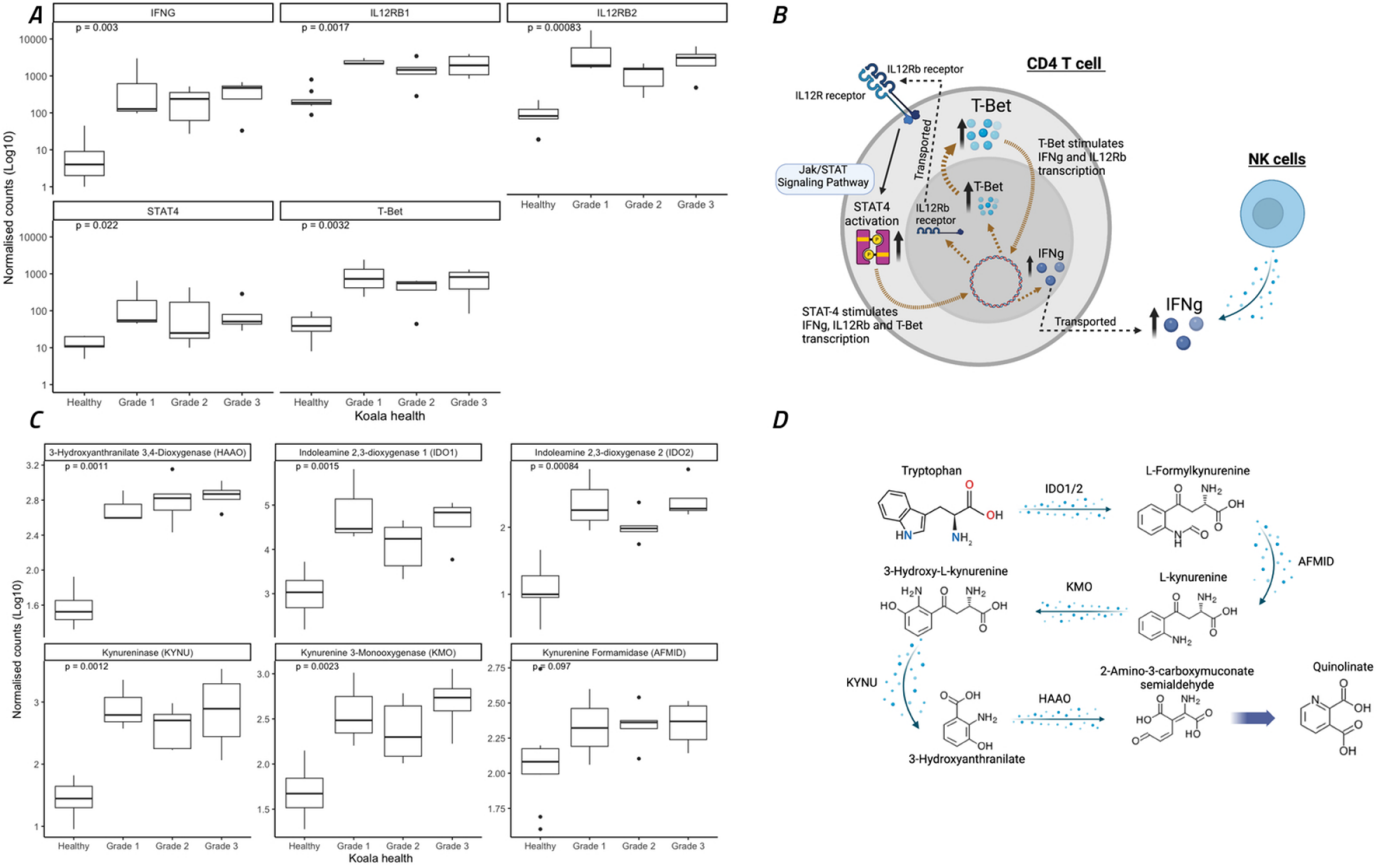
Expression of eleven genes involved in interferon gamma (IFNγ) regulation through Th1 cellular immunity and the Jak/STAT signaling pathway and metabolism of tryptophan to quinolinate through the NAD biosynthesis pathway. **(A, C)** Koala gene expression profiles for eleven gene comparisons assessed by the nonparametric Wilcoxon test, with boxes corresponding to the first and third quartiles (the 25th and 75th percentiles) and whiskers representing the first and forth quartiles. **(B)** The specific pathways for IFNγ expression indicated as significantly different between healthy and diseased koalas (according to KEGG pathway analysis). **(D)** The specific pathways for tryptophane metabolism indicated as significantly different between healthy and diseased koalas (according to KEGG pathway analysis). **(A, C)** were constructed using R studio, **(B, D)** were constructed using Biorender.

All koalas with ocular disease (G1 – G3) had significant (p=0.003) increases in *IFNγ* gene expression compared to healthy animals (**Figure 5B**). The KEGG pathway involving *STAT4* activation, and the Jak/STAT signalling pathway, part of the KEGG pathway ‘Th1 and Th2 cell differentiation’, contained 26 koala DEGs, which significantly contributed to this increase in *IFNγ.* This pathway typically represents a TH1 dominated response involving both NK cells directly and CD4 T-cells (**Figure 5A**). In addition to NK- and T-cell regulation of *IFNγ*, the koalas with ocular disease also showed a significant up-regulation of the transcription factor, *T-bet* (**Figure 5B**).

A key mechanism of *IFNγ* expression in chlamydial infections and disease is the conversion of tryptophan to kynurenine utilising *IDO1/2* through the NAD biosynthesis pathway (Team, R.C, 2018). In the current study, tryptophan metabolism to kynurenine and quinolinate (**Figure 5C**) was significantly upregulated in all koala disease cohorts as compared to healthy controls (**Figure 5D**), involving the genes *IDO1/2*, *AFMID*, *KMO*, *KYNU* and *HAAO* (p = 0.002, 0.001, 0.097, 0.002, 0.001 and 0.001, respectively).

## Discussion

Mechanisms in the development of koala ocular chlamydiosis have been relatively understudied with little evidence to explain why some koalas present with subclinical infections, why others develop mild signs of conjunctivitis and why other koalas progress to chronic keratoconjunctivitis. The current study utilizes a detailed analysis of clinical, bacterial, humoral immune and cellular immune differences between koalas with ocular chlamydiosis to help develop an understanding of the differences between clinical presentations. Findings indicate that subclinical disease is marked by a low infectious load (<200 copies), a low systemic IgG titre (<36,000 end point titre) and a lack of any changes in cellular immune responses (as compared to non-infected animals), as demonstrated within the PCA groupings and no DGE between the healthy groups. Disease progression in koalas with clinical signs of ocular chlamydiosis is defined by increases in chlamydial load during active disease and decreases between chronic active and inactive, with similar trends observed with anti-*Chlamydia* systemic IgG titres and little differences in cellular immunity.

These findings correlate to previous evidence (in human and koala studies) that ocular chlamydial load has an association with the progression from a sub-clinical chlamydial infection (H2) to a state of acute active disease (G1) and further to a state of chronic active chlamydial ocular disease (G2) (Burton et al., 2003; Faal et al., 2006; Wan et al., 2011). Consistent with previous studies (Wan et al., 2011; Nyari et al., 2019), this study found that koalas with chronic inactive disease (G3) had significantly lower *C. pecorum* infectious loads than the chronic active disease cohort (G2; p=0.003; **Figure 4**). This is comparable to trachoma association studies that found individuals with inactive scarring have an association to low-to-undetectable levels of *Chlamydia* (Solomon et al., 2003).

The lack of understanding about the immune response of the koala to *Chlamydia* infection remains a major hurdle in the development of an effective koala-specific *Chlamydia* vaccine (Waugh et al., 2016). The current study utilises RNA sequencing techniques and findings first described in Phillips et al., 2019 from koala urogenital disease (Phillips et al., 2019a) and is the first to provide a comprehensive analysis of the host conjunctival cellular immune response to chlamydial infection across different cohorts of ocular disease.

Chlamydial-induced ocular disease in koalas shares many similarities to the same disease observed in humans. Ocular disease progresses from acute and chronic with a marked increase, then a decrease in infectious load. Based on the results of this koala study, the systemic and mucosal immune response also appears to be relatively predictable, with increases in antibodies, and gene expression of inflammatory cells (NKs, T cells and DCs), specific cytokines (including *IFNγ*) and pathways that influence the metabolism of tryptophan. However, despite this strong anti-chlamydial response, infections in some individual koalas continue to proliferate, with increased inflammation leading to histological changes and eventual scarring of the conjunctival tissues and cornea. The current study provides evidence that in the acute stages of disease (grade 1), Th1 and Th2 immune responses are present through IgG antibodies and increases in IFNγ pathways. However, as the disease progresses to a chronic active disease (G2), infection loads climb and there is a shift in the Th2 systemic response only, marked by moderate increases in systemic anti-*Chlamydia* IgG with no change in Th1 responses (measured by IFNγ related pathways). However, once the disease enters the chronic inactive stage (G3), all responses return to levels similar to the active acute stage (G1) with similar bacterial loads, and systemic IgG levels indicating a return back to a balanced Th1 and Th2 response.

Gene expression microarray profiling studies and recent transcriptome-defining studies have implicated numerous genes and pathways as important in the host immune response to chlamydial infection in koalas (Mathew et al., 2013a; Mathew et al., 2013b; Mathew et al., 2014; Morris et al., 2015; Madden et al., 2018; Phillips et al., 2019a) and humans (Burton et al., 2003; Hess et al., 2003; Schrader et al., 2007; Natividad et al., 2010; Burton et al., 2011). However, a clear understanding of the immunopathological basis of disease and progressive scarring remains elusive (Burton et al., 2015).

Previous human studies of chlamydial ocular disease have highlighted the involvement of a T-cell and natural killer (NK) cell immune response (Natividad et al., 2010). It is also well known that the *IFNγ* response has a major role in anti-chlamydial immunity by starving *Chlamydia* of tryptophan (Nelson et al., 2005), a process also thought to be mirrored in koalas and *C. pecorum* infections (Islam et al., 2018; Quigley and Timms, 2021; White et al., 2021).

As described by Lammas et al., 2000, the IL-12 receptor signalling pathway via the binding of activated CD4 T-cells allows for the phosphorylation of *STAT4* and the upregulation of *IFNγ*, which is a crucial component of cell-mediated immunity against intracellular pathogens (Lammas et al., 2000). Up-regulation of *IFNγ* by NK and T-cell signalling in diseased cohorts was also indicated by Natividad et al., 2010 (although study limitations make definitive claims difficult) (Natividad et al., 2010). Therefore, it was not unexpected to find *IFNγ* significantly upregulated in the current study. However, this finding represents the first evidence-based description of *IFNγ*-related immune responses in koalas with chlamydial-induced conjunctivitis. While T-bet regulation has not previously been recognized in chlamydial ocular disease, it has been indicated in murine urogenital infection trials as a dendritic cell (DC) regulator of *IFNγ* (Rixon et al., 2022). In a murine *T-bet* knockout study, *IFNγ* levels were only marginally affected when stimulated with activated DCs (Lugo-Villarino et al., 2003), suggesting that *T-bet* is not the only transcription factor involved in *IFNγ* regulation. Further investigation into the involvement of *T-bet* in the regulation of *IFNγ* in koala chlamydial ocular disease is required to further understand it’s involvement.

It is well established that the conversion of tryptophan to kynurenine by *IFNγ* is a protective immune response against many bacteria and virus, including *Chlamydia* (MacKenzie et al., 2007). It has also been described in detail that *C. pecorum* have acquired all functional trpABFCDR operons, plus the complementary genes *kynU* and *prsA* allowing them to convert environmental indole from the local microbial community to tryptophan (Ziklo et al., 2016; Ziklo et al., 2018; Hölzer et al., 2020). Therefore, koala chlamydial disease related to *C. pecorum* can bypass NAD biosynthesis pathways through the use of microbial indole and antranilate (Islam et al., 2018). However, there are currently limited reports on the microbiome of the koala conjunctivae, so it is unknown if koala ocular *Chlamydia* infections can avoid tryptophan starvation. From the trachoma literature, a case control study comparing the ocular microbiome in healthy and diseased men and women from Gambia identified differences in the ocular microbiome of people with trachoma, though no causative effect of the microbiome was determined (Zhou et al., 2014). Subsequent studies (also from Gambia) have identified that there is no correlation to the ocular microbiome and trachoma in children but that there were some indications that adults with scarring trachoma had a lowered and altered ocular microbiome. This study identified that an increased abundance of *Corynebacterium* in scarring disease was associated with increased matrix adhesions and a decrease in mucins and may be contributing to scarring (Pickering et al., 2019). *Corynebacterium* are part of the phylum of Actinobacteria which was also indicated as a significant factor in koala ocular disease (Quigley et al., 2018b). It has also been noted that *C. trachomatis* ocular-specific strains have an incomplete trpRBA operon, so the mechanisms of immune avoidance between the two species may differ (Bommana et al., 2021).

There are also known side effects related to increased kynurenine enzymes which may be having a detrimental effect on koalas with chronic ocular chlamydial disease and infections. There is evidence to show that kynurenine enzymes have cytotoxic effects on T-cell regulation by inducing differentiation of naïve CD4+ T-cells to immunosuppressive T-regulatory cells (Kim and Tomek, 2021). Given the importance of CD4+ T-cells in adaptive immunity, this may be one factor effecting immunity to chlamydial infections.

A limitation to this study is the absence of *Chlamydia* DNA sequencing or genotyping. Previous studies have indicated that some strains of *C. pecorum* may be more pathogenic than others in koalas and may be adding to koala disease progression (Robbins et al., 2020). Other limitations are related to sample sizes, and the studies ability to only determine moderate effect sizes.

This study shows key microbial and immunological differences between ocular disease grades in koalas, paralleling aspects of the disease process described in trachoma. This sets a foundation to allow important advances and understanding from trachoma research to be investigated towards koala disease management. Given the critical state of koalas in the wild, any knowledge that can be applied to reducing morbidity and mortality caused by ocular chlamydial infections in koalas will be advantageous to everyone. The findings from this study may be useful in establishing biomarkers of disease progression in koalas and possibly humans. If disease progression can be established prior to increases in tissue inflammation and the resultant scarring can be avoided, this would result in lowering the prevalence of blinding keratoconjunctivitis or trachoma.

## Data Availability

The RNA sequencing data presented in the study are deposited in the SRA database under the BioProject identification number PRJEB26467. This BioProject (PRJEB26467) is part of a larger study that includes 41 bio samples, however only 27 bio samples were utilised for this study, representing koala ocular transcriptomes. The original contributions presented in the study related to Chlamydia and antibody detection are included in the article/supplementary material, further inquiries can be directed to the corresponding author.

## Supplementary material

Supplementary Figure 1 A correlation analysis of chlamydial combined load and anti *C. pecorum* IgG responses reveals a negative correlation between IgG and chlamydial load increases. Shapes represent no infection as circle, ocular and UGT infection as triangle, ocular infection only as square, and UGT infection only as plus sign.; Comparisons assessed by the spearman’s correlation test, Figure was constructed using R studio.
