# Supplementary figures and images for: Koala ocular disease grades are defined by chlamydial load changes and increases in Th2 immune responses
Source: Front Cell Infect Microbiol. 2024 Nov 12;14:1447119. doi: 10.3389/fcimb.2024.1447119 (PMC11588732; doi:10.3389/fcimb.2024.1447119)

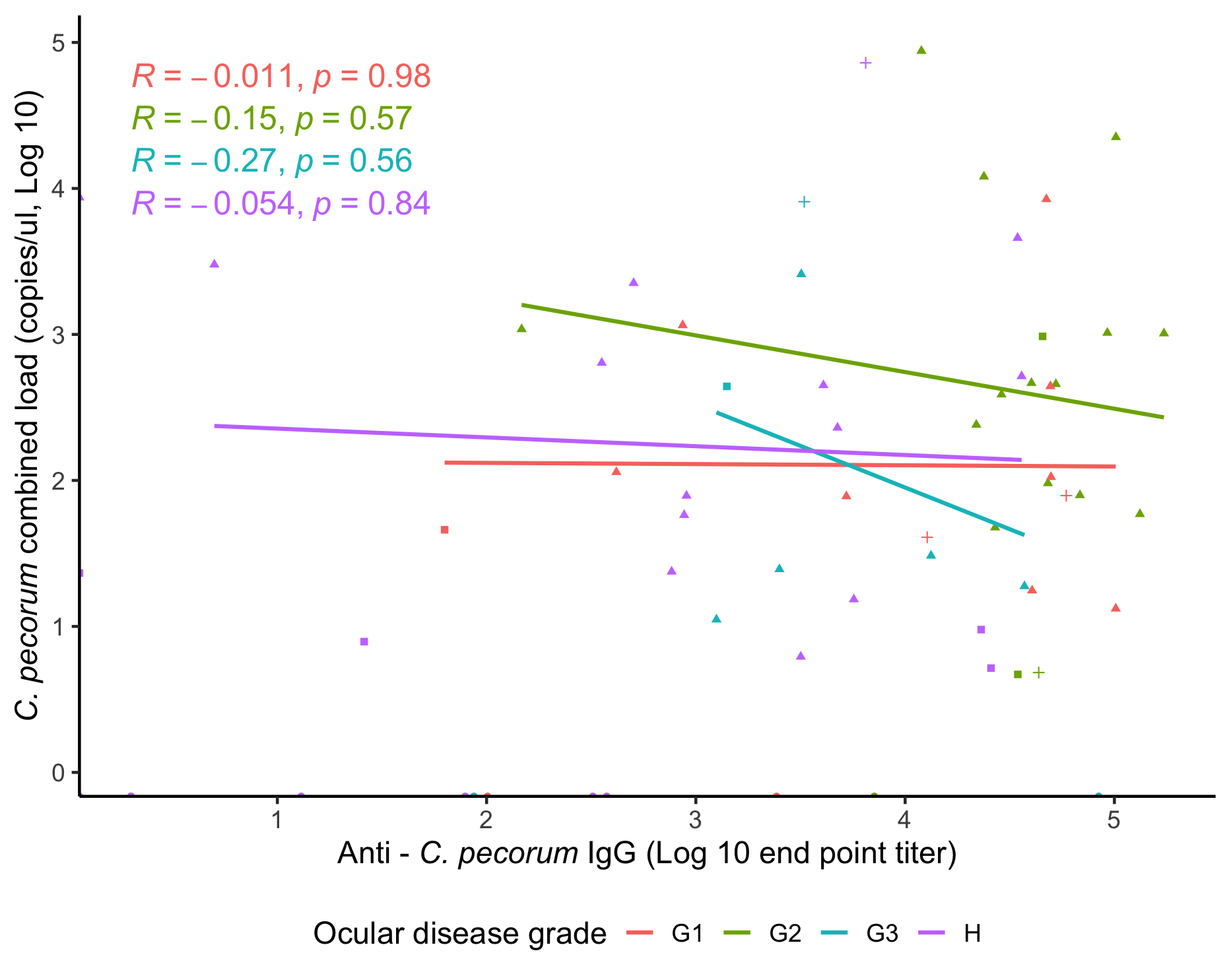

Supplement: Supplementary Figure 1 — A correlation analysis of chlamydial combined load and anti C. pecorum IgG responses reveals a negative correlation between IgG and chlamydial load increases. Shapes represent no infection as circle, ocular and UGT infection as triangle, ocular infection only as square, and UGT infection only as plus sign.; Comparisons assessed by the spearman’s correlation test, Figure was constructed using R studio. [file Image1.png]
